# Violence, Stigma, and Moral Injury in Nursing During the COVID‐19 Pandemic: A Qualitative Analysis From 18 Countries in Latin America and the Caribbean

**DOI:** 10.1111/nin.70153

**Published:** 2026-08-02

**Authors:** Liana Amorim Corrêa Trotte, Marluci Andrade Conceição Stipp, Maria Gefé da Rosa Mesquita, Javier Isidro Rodríguez‐López, Juana Jiménez‐Sánchez, Sandra Milena Aponte‐Franco, María Guadalupe Casales‐Hernández, Gabriela Castillo‐Veras, Paola Andrea Dixon, Julio Alejandro García García‐Castillo, Monaliza Gomes Pereira, Marcella Gabrielle Betat, José Luís Guedes dos Santos, Allison Squires

**Affiliations:** ^1^ Anna Nery School of Nursing Federal University of Rio de Janeiro Rio de Janeiro Brazil; ^2^ Growing Up Foundation; ^3^ Department of Nursing Federal University of Santa Catarina Florianópolis Brazil; ^4^ Universidad Autónoma Metropolitana – Xochimilco Mexico City Mexico; ^5^ Universidad Autónoma de Santo Domingo Santo Domingo Dominican Republic; ^6^ Universidad de Carabobo Valencia Venezuela; ^7^ Rory Meyers College of Nursing New York University New York New York USA

**Keywords:** COVID‐19, Latin America, moral distress, moral injury, nurses, workplace violence

## Abstract

Workplace violence against nurses and midwives intensified during the COVID‐19 pandemic, exposing and amplifying longstanding structural marginalization in health systems across Latin America. This study analyzes how different forms of violence were experienced and described by nursing professionals during this period. A secondary qualitative analysis was conducted using text‐based responses from an international online survey developed by the Global Consortium, which included 3860 nurses and midwives from 18 Latin American and Caribbean countries. Data were examined through Descending Hierarchical Classification using IRaMuTeQ, followed by reflective thematic analysis for interpretive integration. The analysis identified five lexical classes, reorganized into three interconnected themes: direct violence related to care delivery and system collapse; stigma, fear, and disruption of social belonging; and ethical–political suffering associated with structural blame, moral suffering, and moral injury. Despite contextual differences across countries, narratives revealed recurring patterns of emotional overload, professional delegitimization, and exposure to multiple forms of violence. These findings position violence against nursing professionals during the pandemic as a structural and multifaceted phenomenon rooted in historical inequities and institutional fragility, underscoring the need for sustained policies that strengthen workforce protection, ethical accountability, and institutional support in both routine healthcare settings and future public health emergencies.

## 1 Introduction

The COVID‐19 pandemic placed unprecedented pressure on health systems, increasing the workload and exposure of health professionals, especially nurses, to physical, emotional, and social risks (Groves et al. 2023; Saragih et al. 2022). The crisis has intensified occupational vulnerabilities, raising levels of violence, exhaustion, and mental distress among health professionals (Zhang et al. 2023). Care overload, fear of contagion, resource shortages, and social tension have created a hostile environment in which nurses have been among those most exposed to verbal, emotional, and physical aggression (Eshah et al. 2024).

Violence against nursing professionals, recognized by the World Health Organization (WHO) and the International Council of Nurses (ICN) as a serious occupational health problem, intensified during the COVID‐19 pandemic. Estimates indicate that between 8% and 38% of healthcare workers have experienced physical violence, in addition to frequent exposure to verbal abuse and threats (International Labour Organization et al. 2002). ICN reports indicate that about 60% of professionals observed an increase in these incidents at the beginning of the pandemic, highlighting the worsening of social and institutional tensions in health services (International Council of Nurses 2022).

Despite the increasing international scientific output on violence against nurses during the COVID‐19 pandemic, significant gaps remain in understanding these experiences as they occurred in Latin America, a region marked by vast structural inequalities, fragile health systems, and historically precarious working conditions (Bustamante and Méndez 2014; Macinko et al. 2016). Examining these specificities through a transnational approach is essential to inform health policies and institutional responses that are sensitive to local sociopolitical and structural contexts, both during public health emergencies and in routine healthcare settings, while addressing persistent forms of violence against nurses that extend beyond crisis situations.

In May 2025, the World Health Assembly adopted the WHO Pandemic Agreement (WHA78.1), establishing a global normative framework for pandemic preparedness, health system resilience, and health workforce protection (World Health Assembly 2025). Articles 6 and 7 emphasize strengthening health systems and protecting the health workforce, underscoring the central role of nursing professionals. In this context, empirical evidence on violence, stigma, and moral suffering among nurses is essential to inform the implementation of these provisions at the country level. Accordingly, this study examines how workplace violence shaped the experiences of Latin American and Caribbean nurses during the COVID‐19 pandemic.

## 2 Background

### 2.1 Extent and Forms of Violence in Nursing

Workplace violence against healthcare professionals was a globally recognized and highly prevalent issue long before the emergence of COVID‐19. A meta‐analysis conducted immediately before the pandemic reported that 61.9% of healthcare professionals had experienced some form of workplace violence. Verbal violence was the most common manifestation, affecting 57.6% of workers, followed by physical violence (24.4%) and sexual harassment (12.4%) (Li et al. 2020). Similar findings were reported in a review involving nurses from Southeast Asia and the Western Pacific, where rates of physical violence ranged from 14% to 34% and verbal abuse from 59% to 70% (Varghese et al. 2022). These data, already recognized as evidence of a serious occupational health problem (Liu et al. 2019; Sahebi et al. 2022), provide an important baseline for understanding how the COVID‐19 pandemic amplified and made more visible preexisting forms of workplace violence (Hadavi et al. 2023).

In health services, workplace violence is a complex phenomenon encompassing incidents in which professionals are subjected to abuse, threats, or aggression arising from their professional practice, directly or indirectly compromising their physical and psychological integrity. Beyond immediate harm, such violence poses a structural threat to nurses' well‐being, defined as a positive state shaped by social, economic, and environmental conditions that support quality of life, sense of purpose, and collective capacity for resilience and equitable action, as described by the World Health Organization (2021) and highlighted in recent analyses of nurse well‐being and work environments (Bond et al. 2025; Çakir et al. 2025).

Within this framework, the taxonomy of workplace violence fundamentally distinguishes physical and psychological forms. Physical violence involves the use of force that results in, or is likely to result in, physical, sexual, or psychological harm, whereas psychological violence is characterized by the deliberate use of power, including explicit or implicit threats, that undermines mental, moral, social, or spiritual integrity (International Labour Organization et al. 2002). Verbal abuse, harassment, and intimidation are prevalent manifestations of psychological violence in healthcare settings and have been associated with anxiety, depression, burnout, and impaired professional engagement among nurses, with important implications for mental health and moral well‐being.

Beyond these widely recognized modalities, the literature has identified less visible but equally consequential forms of violence, including symbolic violence and moral violence. Symbolic violence consists of practices of delegitimization, stigmatization, and disqualification that become naturalized in everyday life, eroding workers' social and professional recognition (Bourdieu and Wacquant 1992). Moral violence is analytically linked to the concepts of moral distress and moral injury. In this article, moral distress refers to the suffering that occurs when professionals recognize the ethically appropriate course of action but cannot act due to external, organizational, or structural constraints (Morley et al. 2019), while also acknowledging the theoretical tensions highlighted by Varcoe et al. (2012). Moral injury denotes more enduring harm to moral identity and integrity resulting from potentially morally injurious events, such as being compelled to act against one's core values or being unfairly blamed for systemic failures (Čartolovni et al. 2021; Rabin et al. 2023). Building on these concepts, we use the term moral violence to describe forms of institutional and relational violence that undermine nurses' moral agency by silencing ethical concerns, constraining ethically motivated action, or shifting responsibility for structural problems onto individuals, thereby producing moral suffering and compromising ethical integrity.

Engaging with broader debates on moral reconstruction in contexts of social injustice and structural wrongs, we define moral reconstruction as the processes by which nurses and collectives work to rebuild moral frameworks and professional identities after experiencing moral violence and moral injury, rearticulating shared meanings of good care, responsibility, and justice (Young 2011; Roht‐Arriaza and Mariezcurrena 2006). Similarly, drawing on critical accounts of moral domination in social and political theory, we use this term to describe the asymmetrical imposition of moral norms and meanings by healthcare institutions, where managerial or policy agendas override or silence nurses' ethical perspectives and normalize unjust blame for structural failures (Fraser 2010; Samuel 2012). These concepts allow workplace violence to be understood not only as a series of discrete events but also as a moral field in which power relations determine whose values matter, whose voices are heard, and how nurses can or cannot reconstruct their ethical agency.

These forms of violence intensified during the COVID‐19 pandemic, particularly among nurses working under conditions of extreme resource scarcity, institutional instability, and emotional overload (Scoglio, Stelson, et al. 2024). These factors broaden the understanding of workplace violence beyond physical or verbal aggression by highlighting mechanisms of symbolic domination, stigma, and unfair blame (Saragih et al. 2022). In this context, the notion of ethical–political suffering is useful for capturing the suffering produced by inequalities, power relations, and institutional mechanisms that disempower professionals' ethical agency and erode their capacity to act in accordance with their moral commitments (Capella 2022).

Exposure to workplace violence seriously affects nurses' mental health, leading to anxiety, depression, burnout, and sleep disorders, which compromise both well‐being and professional performance (He et al. 2026; O'Brien et al. 2024). These effects impact the quality and safety of care, as psychologically distressed and morally injured nurses have greater difficulty sustaining therapeutic relationships, clinical vigilance, and ethical deliberation in direct care settings (Agbornu et al. 2022; Connell et al. 2026). At the organizational level, workplace violence is linked to increased absenteeism, turnover, and intentions to leave the profession, resulting in the loss of experienced nurses and higher institutional costs (Banga et al. 2023; O'Brien et al. 2024). Together, these dynamics reinforce the recognition of workplace violence as a critical threat not only to individual nurses but also to the sustainability, resilience, and justice of health systems.

### 2.2 Gaps in the Literature and Rationale for the Study

Despite the increasing number of studies on violence in healthcare work, there is a lack of research that captures the experiences of nurses in Latin America and Caribbean. Most studies about nurses' experiences with violence are focused on high‐income countries, which limits understanding of the regional specificities and sociocultural dynamics that shape how violence manifests in nursing practice in the region (Banga et al. 2023; Zhang et al. 2023; O'Brien et al.^2024^).

Few studies specific to the COVID‐19 pandemic and nurses' experiences with violence exist that are specific to Latin America and Caribbean. In Brazil, inequalities among health professions have increased, with nurses reporting poorer working conditions, greater exposure to infection risk, and insufficient institutional protection (Bitencourt et al. 2021). At the regional level, a multicenter study involving 18 Latin American countries found that 68.6% of nurses experienced some form of aggression during the pandemic, highlighting how preexisting vulnerabilities became direct hostility at work (Gupta et al. 2024). However, this study has limitations, such as data collected only between January and February 2022 and about three‐quarters of the sample concentrated in Argentina, Ecuador, and Mexico, which reduces the region's heterogeneity and underrepresents countries with greater institutional precariousness and fragility.

Despite growing concern about workplace violence against nurses, there remains a lack of multinational qualitative synthesis capturing how violence, stigma, and ethical–political suffering are experienced and articulated across Latin American and Caribbean contexts. In particular, there is limited interpretive integration of large‐scale narrative data capable of revealing shared and divergent patterns of meaning across countries.

## 3 Aim of Study

To analyze the experiences of nurses from 18 countries in Latin America and the Caribbean who were exposed to various forms of violence during the COVID‐19 pandemic.

## 4 Methods

### 4.1 Study Design

This study is a secondary analysis of data collected through an online descriptive qualitative investigation conducted by the Global Consortium of Nursing & Midwifery Studies (GCNMS), an international collaborative research network dedicated to advancing nursing and midwifery knowledge. The broader study aimed to document, through participants' narratives, the experiences of nurses and midwives working in healthcare services providing care to patients with COVID‐19 across various global contexts during both the intra‐pandemic and post‐pandemic phases.

Qualitative secondary analyses draw data from a primary or parent study to answer a new question (Ruggiano and Perry 2019;). For international comparative studies, they can allow for comparisons of how contextual differences manifest within the same phenomenon and also where similarities arise (Hughes et al. 2023). It is considered a useful approach for making policy recommendations related to healthcare delivery (Ziebland and Hunt 2014). Reporting follows the COREQ guidelines from the Equator Network for qualitative studies.

### 4.2 Population and Sample

The total population in this data sample, drawn from the international consortium, included participants (nurses and midwives) residing in 18 countries in Latin America and the Caribbean. However, Cuba and Haiti were excluded, so the sample comprised participants from the remaining countries. To participate in the study, individuals had to be nurses or midwives who provided direct care to patients diagnosed with COVID‐19 at any time between March 2020 and December 2023. Those enrolled in basic educational programs were excluded. In this sample, respondents who answered “yes” to the question, “During the last two years, as a nurse or midwife, have you experienced any anger or aggression from the public?” and provided a narrative description of their experience in the open‐ended response field were considered eligible.

As this is a descriptive study with a qualitative approach, no minimum sample size was established. The main inclusion criterion was the presence of a narrative report with at least four words forming a coherent phrase as this was sufficient to allow lexical analysis by the software used to analyze them.

### 4.3 Instrument

The parent study's questionnaire was developed by the consortium and covered seven key areas: mental health, work environment, economics, migration, care management, occupational risks, and a comprehensive sociodemographic profile. In the absence of standardized instruments for the phenomenon under investigation, the questions were developed based on available evidence and finalized through a modified Delphi process with expert consensus. The instrument was originally developed in English and later translated into Spanish and Portuguese (Brazil) using standardized procedures previously described in the literature (Squires et al. 2013).

The open‐ended question analyzed in this study was part of the mental health section and was formulated as follows: “During the last two years, as a nurse or midwife, have you felt any anger or aggression from the public?” The response options were: (1) Yes—describe in the space below; (2) No; (3) I do not know; and (4) I prefer not to say. All responses were stored in a secure electronic repository with restricted and encrypted access, in accordance with international ethical standards for data protection.

### 4.4 Data Collection

Data collection occurred from July 2022 to May 2025, covering the final period of the COVID‐19 pandemic and the following 2 years. Recruitment was carried out through social media, international listservs, professional associations, and existing international nursing and midwifery collaboration networks, with country team members leading local recruitment efforts. Participation was further expanded using a snowball recruitment strategy, in which nurses, midwives, researchers, or professional representatives already involved in the study invited colleagues and professional contacts from other countries to participate and coordinate local dissemination.

Data collection was conducted in multiple languages using a shared online survey link. Teams were encouraged to recruit at least 300 participants per country during the data collection period. All participating teams received access to data specific to their respective countries for independent analysis and dissemination.

Eligible participants were nurses and midwives who worked during the COVID‐19 pandemic and voluntarily agreed to participate in the study. No formal exclusion criteria were established.

The global questionnaire was hosted on the primary study's Qualtrics XM Survey platform, selected for its security, linguistic adaptability, and multicenter management capabilities. Access to the instrument was provided through an exclusive electronic link distributed in participating countries via social networks, professional associations, educational institutions, and healthcare settings, as permitted within each country.

Recruitment strategies relied on the social and professional networks of national study collaborators and were supplemented by dissemination through digital platforms, professional organizations, and email invitations. Each national team established its own data collection schedule, ranging from a minimum of 4 months to a maximum of 12 months.

Data collection was completely anonymous. The system did not record names, email addresses, or other personal identifiers. IP addresses were automatically anonymized, preventing response traceability. The platform also blocked multiple submissions from the same IP address, reducing the risk of duplicate responses and automated interference (bots). Each participant received a unique alphanumeric identifier used exclusively for internal quality control and data integrity verification.

Online dissemination through professional and institutional networks was adopted due to pandemic‐related restrictions and the multicenter nature of the study. The researchers had no prior relationship with the participants. Likewise, participants had no information about the researchers beyond the general study description provided in the informed consent form at the time of data collection.

### 4.5 Ethical Aspects

The parent study was approved by the New York University Institutional Review Board (IRB‐FY2020‐4440). New York University was the institution that established the Global Consortium and served as the IRB of record for all participating country partners. Costa Rica obtained additional approval from the National Ethics Committee. All participants signed an informed consent form prior to the initiation of data collection. Since this study aligned with the analytic aims of the parent study, it did not require separate approval from the parent study's home institution (Irwin et al. 2012).

### 4.6 Data Analysis

Demographic responses associated with the free‐text data were extracted from the Qualtrics platform in comma‐separated values (CSV) format and processed using R statistical software (version 4.1.2). Continuous variables, such as age, were analyzed using mean, median, and standard deviation. Categorical variables were described using absolute frequencies and percentages. Descriptive analyses were performed for the total sample and for each country. The sample characterization provided a basis for contextualizing the subsequent qualitative stage.

The study adopts an interpretive framework, viewing the written narratives produced in the open questions of the online questionnaire as situated constructions of meaning, developed from participants' lived experiences in specific institutional, social, and historical contexts. Although mediated by a self‐administered instrument, these narratives express subjective interpretations of the phenomenon under investigation and allow analysis of the meanings attributed to the reported experiences.

The open‐ended responses linked to the Global Consortium database underwent spelling standardization, anonymization, and semantic equivalence review between Spanish and Portuguese, conducted by the national teams. The analytical process is described in detail in Supporting Information S1: Table 1. As this is secondary data from a single open‐ended question in a large‐scale international survey, the analytical objective was not to achieve theoretical saturation but to identify recurring discursive patterns that were sufficiently stable to support a multinational interpretive synthesis. Furthermore, since data were collected over a 3‐year period, this approach allowed management of potential changes over time and ensured consistency in the analytic process (Irwin et al. 2012).

The narratives were organized into 18 plain text files (one per country) and combined into a single corpus after textual cleaning. The material was processed using IRaMuTeQ software, segmented into Elementary Context Units (approximately 40–50 words), and analyzed using Descending Hierarchical Classification, derived from the ALCESTE software (Souza et al. 2018). This technique groups text segments into lexical classes based on word frequency and statistical significance, identifying clusters of shared meaning. Class formation relied on chi‐square (*χ* ^2^) tests, and a minimum classification rate of 75% was adopted to ensure analytical reliability (Rodríguez Rodríguez et al. 2024; Souza et al. 2018).

The interpretive stage followed Reflective Thematic Analysis (RTA) using an inductive approach, including iterative reading of classes, initial coding, review of internal coherence, and thematic synthesis. This process was conducted by six experienced PhD‐level researchers of different genders through regular analytical meetings with peer debriefing (Braun and Clarke 2022). The integration of DHC and RTA combined lexical structure with narrative interpretation, resulting in three analytical themes: direct violence, social stigma, and ethical–political suffering.

Data were collected in Spanish and Portuguese. Initial familiarization with the data, line‐by‐line coding, and preliminary theme development were conducted in the original languages by bilingual PhD‐level researchers fluent in both Spanish and Portuguese. Coding frameworks were first developed separately for each language, then compared and refined in joint analytic meetings to ensure semantic and conceptual equivalence across the Spanish‐ and Portuguese‐language datasets.

To address linguistic nuances, the team used a consensus approach: when expressions or idioms lacked direct equivalents in the other language, researchers discussed their contextual and moral meanings, prioritizing conceptual over literal equivalence in constructing codes and themes. The final thematic structure was agreed upon in Spanish and Portuguese before any translation into English. English labels for themes and subthemes, as well as illustrative quotes, were produced through a two‐step process: initial translation by a bilingual researcher experienced in qualitative research and academic writing in English, followed by independent review and minor adjustments by another bilingual team member, with particular attention to preserving ethical and emotional connotations rather than strict word‐for‐word correspondence.

This analytical process ensured rigor through the transparent integration of Descending Hierarchical Classification and Reflective Thematic Analysis, with explicit documentation of analytic parameters, iterative reading of lexical classes, initial coding, and review of internal coherence. Reflexivity was addressed through ongoing critical discussions within the research team during the interpretive stage, supporting a careful thematic synthesis that articulated direct violence, social stigma, and ethical–political suffering across national contexts.

### 4.7 Participant Characteristics

This research included nurses and midwives from 18 Latin American and the Caribbean countries. The parent survey had 6985 respondents: 6703 nurses and 283 midwives. Of these, 3851 (55%) of professionals reported experiencing some type of violence during the COVID‐19 pandemic, forming the analytical population described. Nursing professionals predominated (*n* = 3686; 96.0%) compared to midwives (*n* = 165; 4.0%), and the group was predominantly female (*n* = 3122; 81.1%). The average age was 45.0 years (SD ± 16.3).

Participants were distributed across multiple countries, with the largest proportions from Guatemala (14.1%), Paraguay (12.6%), and El Salvador (9.9%). Most participants worked in hospital services (*n* = 2220; 71.1%) and in urban areas (*n* = 2515; 80.9%), with significant representation in intensive care units (*n* = 606; 19.4%), emergency services (*n* = 550; 17.6%), and primary, community, or outpatient care (*n* = 610; 19.5%). The majority worked direct service care roles (*n* = 2192; 67.3%) and were employed full‐time (*n* = 1733; 55.5%). The total number of participants varies by variable due to missing responses in some categories. A summary of participants' demographic and professional characteristics is presented below. Detailed participant characteristics are provided in Supporting Information: Table 2.

## 5 Thematics Findings

The DHC analysis resulted in five stable lexical classes with a relatively homogeneous distribution of text segments across themes. Classes 1 and 3 were the most representative, each accounting for 20.6% of the corpus, followed closely by Class 2 (20.4%) and Class 5 (20.0%). Class 4 represented the smallest proportion, comprising 18.4% of the corpus. This balanced distribution indicates a cohesive lexical structure, with distinct yet comparably weighted semantic domains. A graphical representation of the lexical distribution is provided in Supporting Information S3.

During the interpretative stage using RTA, strong semantic convergence was observed between Classes 1 and 3, and between Classes 2 and 4, which justified their reorganization into two integrated themes. Class 5 was maintained as an independent theme due to its distinct ethical–political character. This reorganization resulted in three major analytical themes. Illustrative quotations supporting each analytical theme are presented in Table 1.

**Table 1:** Reflective themes—Violence and suffering among Latin American and Caribbean nursing professionals during the COVID‐19 pandemic.

Central theme	Emerging subthemes	Representative countries	Typical UCEs^a^ (literal statements)	Atypical UCEs (literal statements)	Reflective interpretation
1. Direct violence in care and system collapse (Classes 1 + 3)	Verbal and physical aggression; Overload and social impatience; Social overload and impatience; Collective anger and structural failures; Undue accountability for staff	Chile Brazil Colombia Peru Costa Rica Paraguay Bolivia	“In the emergency room, patients ignore the triage system and demand immediate attention” (Chile). “We experience verbal and physical abuse almost every day; waiting is the main reason” (Brazil). “Families direct their anger at the team, blaming us for the lack of resources” (Colombia). “Overcrowding and understaffing cause aggression” (Chile). “The lack of oxygen resulted in constant insults and threats” (Peru).	“Some family members apologized when they realized how desperate the situation was” (Mexico). “With empathy and dialogue, some of the anger was contained” (Chile).	This theme highlights the relational collapse of care: Violence arises from the intersection of structural fragility, social suffering, and professional overload. The presence of empathy in some contexts shows that relational mediation can help contain conflicts.
2. Stigma, fear, and the breakdown of social belonging (Classes 2 + 4)	Public stigma and fear of contagion; Community exclusion; Family isolation; Identity distress	Brazil Chile Honduras Mexico Guatemala Bolivia	“I was prevented from using the elevator because they thought I was carrying the virus” (Brazil). “People shouted that we were spreading COVID‐19” (Chile). “My neighbors threw bleach at me out of fear” (Honduras). “We were prevented from entering the city, accused of carrying the virus” (Guatemala). “My mother separated my plate out of fear of contamination” (Brazil).	“Some patients recognized our efforts and lamented the prejudice” (Peru). “Over time, some of the population began to appreciate our work” (Uruguay).	The theme reveals the symbolic violence of stigma, which extends beyond the workplace and affects family and community life. The breakdown of social belonging weakens professional identity and intensifies moral suffering. Atypical statements indicate pockets of solidarity that reconfigure social recognition.
3. Ethical–political suffering and structural blaming (Class 5)	Lack of supplies and beds; Moral blame of the nurse; Moral blame of nurses; Symbolic institutional violence; Extreme precariousness	Bolivia Brazil Chile Colombia Venezuela	“How can we not have medicines if you are health professionals?” (Bolivia). “The lack of PPE led to insults from the community” (Brazil). “The population was aggressive due to the lack of consultations and long waits” (Chile). “We were accused of negligence for deaths related to the lack of oxygen” (Colombia).	“Some patients understood our limitations and tried to cooperate” (Ecuador). “Some family members expressed gratitude, despite the lack of resources” (Paraguay).	This theme expresses the ethical and political core of moral suffering: Nurses are held responsible for structural failures beyond their control. In countries with weaker healthcare systems, the intensity of blame increases. Even so, gestures of understanding emerge that reaffirm the ethical resilience of care.

a UCE = Elementary Context Unit.

### 5.1 Direct Violence in Care and the Collapse of the System (Classes 1 and 3)

Direct violence in care and the collapse of the system (Classes 1 and 3) brings together reports of verbal and physical aggression, social impatience, care overload, and the unfair accountability of the nursing team for structural failures in health services. The discourses show that the care environment has become a space of constant tension, marked by long queues, resource shortages, and misinformation, factors that have triggered recurrent episodes of hostility, mainly directed at nurses, who are the most visible professionals in the care chain. Even so, there are reports of empathy, mediation, and conflict containment, revealing professionals' relational capacity even under extreme pressure.

### 5.2 Stigma, Fear, and Rupture of Social Belonging (Classes 2 and 4)

Stigma, fear, and rupture of social belonging (Classes 2 and 4) describes the symbolic transformation of nurses into perceived sources of contamination. The fear of contagion led to rejection in public and community spaces and eventually entered the home, causing emotional isolation, humiliation, and a loss of belonging. This symbolic violence weakened professional identities and social bonds, although in some contexts, experiences of solidarity partially mitigated the stigma.

### 5.3 Ethical–Political Suffering and Structural Blaming (Class 5)

Ethical–political suffering and structural blaming (Class 5) reveals a deeper dimension of moral suffering, linked to blaming professionals for shortages of supplies, beds, oxygen, and personnel. In contexts of greater institutional precariousness, this transfer of responsibility intensifies, making nurses the “visible face of collapse.” Even so, narratives of recognition and cooperation emerge, highlighting the persistence of an ethic of care even in scenarios of extreme scarcity.

In general, the three themes reveal a pattern of structural marginalization, social stigma, and moral suffering that spans various contexts of Latin American nursing during the pandemic. Together, the three themes outline a continuum of violence and suffering that spans both healthcare settings and private life, simultaneously revealing the wounds and the ethical and social strength of Latin American nursing. Despite national differences, the narratives converge on recurring experiences of daily violence, emotional overload, and professional delegitimization. Although the intensity of these processes varies among countries, with greater empathy reported in Chile and Mexico and more pronounced rejection in Honduras, Guatemala, and Venezuela, this convergence indicates that violence against nursing extends beyond local specificities, constituting a structural expression of longstanding fragilities in public health systems across the region, which were intensified and rendered more visible during the pandemic.

## 6 Discussion

This study is one of the first systematic qualitative analyses of violence experienced by nurses in Latin America during COVID‐19, revealing a regional pattern that has been little documented until now. The reports indicate that violence and moral suffering resulted from a combination of care overload, social stigma, and structural accountability. The synthesis of lexical classes into three themes shows that these experiences occurred in interdependent layers of relational, identity, and political‐institutional vulnerability, shaped by conditions historically rooted in the region's health systems. The innovative approach demonstrated the persistence of these themes across 18 countries and denotes the structural commonalities that drove Latin American nurses' and midwives' COVID‐19 pandemic work experiences.

Despite national differences, the narratives converge in describing daily violence, emotional exhaustion, and professional delegitimization. This regional pattern suggests that violence against nurses is not an episodic phenomenon, but rather a persistent structural phenomenon rooted in longstanding inequities in the organization of Latin American and Caribbean health systems, which became more visible under pandemic conditions (Lotta et al. 2021). The qualitative findings also contextualize the quantitative results of previous studies on the topic (González‐González et al. 2025; Gupta et al. 2024).

The findings concerning direct violence in care and the collapse of the system reveal that verbal and physical aggression were manifestations of broader structural failures, arising from the convergence of care overload, misinformation, and institutional precariousness. The shortage of staff, beds, and supplies, along with chaotic workflows and long waits, made nurses the primary targets of public frustration, a dynamic similar to the “pressure cooker” model described in the literature (Pagnucci et al. 2022; Acosta Nuñez et al. 2025; O'Brien et al. 2024).

During the pandemic, nurses became an intensified target for collective frustration surrounding longstanding deficiencies in healthcare access, infrastructure, and institutional responsiveness. This trend aligns with evidence linking chaotic environments, resource shortages, and emotional overload to increased occupational violence and moral distress (Lee et al. 2024; Squires et al. 2026).

Even so, episodes of empathetic mediation emerged, serving as symbolic containment of violence. In countries such as Chile and Mexico, stronger institutions and improved care flows supported communication practices that reduced conflicts (Fronteira et al. 2024), consistent with study highlighting organizational stability and trust in the system as protective factors (Fahim et al. 2025). At the same time, counter‐narratives of recognition and gratitude have emerged, revealing social ambivalence characterized by the coexistence of fear and appreciation (Fahim et al. 2025).

In addition to physical aggression, the reports reveal an ethical and emotional dimension of violence. That was reflected in the ongoing relational strain between professionals and users, a phenomenon consistent with findings that link occupational violence to institutional collapse and moral suffering (Norman et al. 2021).

The findings concerning stigma, fear, and the rupture of social belonging reveal that fear of contagion socially repositioned nurses from trusted caregivers to perceived vectors of disease, producing experiences of rejection, exclusion, and profound disruption of their professional and social identities. This pattern was also seen in other countries during COVID‐19, where professionals were avoided, harassed, or viewed as health threats (Fronteira et al. 2024).

In some contexts, effective communication strategies and increased institutional support partially mitigated this stigma (Fahim et al. 2025). In the Latin American and Caribbean countries analyzed, however, structural inequality, overburdened health systems, and inadequate public communication intensified the process, turning nurses into symbolic markers of risk and increasing their social isolation (Gupta et al. 2024). International evidence reinforces this pattern: in Nepal, professionals were treated as “untouchables”; in Singapore, healthcare workers reported being evicted from their homes due to fears of COVID‐19 transmission; in Ghana, a nurse was evicted for treating patients with COVID‐19 (Nashwan et al. 2022; Fahim et al. 2025; Squires et al. 2025).

Stigma has also entered the intimate sphere of nurses' homes, causing emotional distancing, domestic surveillance, and feelings of moral contamination among family members, as shown by recent studies. This intrusion into private space represents an intensified form of symbolic violence that erodes professional identities and increases moral suffering (Fahim et al. 2025).

Vulnerability to stigma was further influenced by characteristics of nursing education in Latin America, which has historically focused on technical levels and received lower social recognition, weakening professional authority (Cassiani et al. 2017). In Honduras, for example, nurses report being seen as assistants with no authority, which exacerbates community mistrust in crisis contexts (Hwang 2025).

The findings related to ethical–political suffering and structural blaming suggest that nurses' suffering is produced not by individual vulnerability, but by institutional arrangements that normalize precariousness and shift responsibility for structural failures onto individual professionals. Operating simultaneously at material, symbolic, and political levels, these dynamics constitute the deepest expression of institutional violence identified in this study. In this context, nurses are held morally responsible for systemic failures beyond their control, a phenomenon that reflects ethical–political suffering. When working conditions make it impossible to ensure safety and minimum quality, yet society continues to demand optimal performance, forms of moral injury arise. These are characterized by a sense of institutional betrayal and the violation of professional values (Norman et al. 2021; Scoglio, Nishimi, et al. 2024).

The structural lack of supplies, beds, and resources is not only a material obstacle but also a mechanism that shifts systemic responsibilities to individuals, reinforcing moral domination (Silva et al. 2023; Eshah et al. 2024). In countries with chronic underfunding, institutional instability, and historical inequalities, this process intensifies (Lotta et al. 2021; Nigenda et al. 2025). There is a clear need to invest in actions to improve working conditions, particularly regarding adequate resources for care and expanding opportunities for managers to enable the nursing team to actively participate in hospital discussions and decision‐making processes (Paula et al. 2024). This dynamic creates fertile ground for moral injury, fueled by powerlessness in the face of scarcity and public exposure as the person held responsible for negative outcomes resulting from structural collapse (Norman et al. 2021; Scoglio, Stelson, et al. 2024).

Despite this adverse scenario, some studies find an ethical countermovement emerging as patients and family members apologize, acknowledge institutional limitations, or value the team's efforts (Carvalho et al. 2023). These gestures restore the possibility of moral reconstruction and reaffirm the ethical dimension of care.

### 6.1 Strengths and Limitations

This study has limitations that should be considered when interpreting the findings. First, convenience sampling and online recruitment via professional networks limited national representativeness and may have overrepresented professionals with greater digital access and engagement. Although this strategy enabled broad regional coverage across 18 Latin American and the Caribbean countries, it restricts country‐level comparisons; therefore, findings are interpreted at a structural and regional level.

Second, the extended data collection period (2022–2025), referring to experiences beginning in 2020, introduces potential recall bias and retrospective reinterpretation. This temporal breadth strengthened the inclusion of multiple pandemic phases but required an analytical focus on shared meanings rather than temporal trajectories.

Third, as a qualitative secondary analysis, the study could not capture longitudinal trajectories of violence or moral suffering. Despite rigorous procedures to preserve conceptual equivalence across languages, some cultural and idiomatic nuances may not have been fully transferable into English. These factors do not compromise the interpretive analysis, which prioritized discursive patterns over representativeness.

Finally, Descending Hierarchical Classification favors dominant lexical structures and may underrepresent minority or intersectional experiences. Nevertheless, the large multinational corpus and systematic analytical approach strengthen the robustness and policy relevance of the findings, supporting their contribution to discussions on workforce protection, psychosocial support, and institutional accountability.

### 6.2 Recommendations for Nursing and Health Policies

Based on the findings, the following recommendations are proposed for nursing and health policies at national and international levels, in alignment with Articles 6 and 7 of the WHO Pandemic Agreement (World Health Assembly 2025).

First, health systems should institutionalize workforce protection as a core component of health system governance and preparedness, rather than as an ad hoc response limited to public health emergencies. This includes formally integrating violence prevention, mental healthcare, and psychosocial support for nurses and midwives into emergency preparedness plans and routine health system governance.

Second, governments and health authorities should adopt explicit policies to prevent and address workplace violence against nursing professionals, including mechanisms for reporting, monitoring, and accountability at both institutional and system levels. These policies must recognize violence, stigma, and moral injury as occupational risks with ethical and legal implications.

Third, nursing leadership and regulatory bodies should strengthen ethical governance and risk communication frameworks, ensuring that nurses are not positioned as symbolic buffers for systemic failures or subjected to institutional blame during health emergencies and in routine healthcare delivery.

Finally, international organizations and national policymakers should use the WHO Pandemic Agreement as a normative tool to operationalize workforce protection, translating its provisions into concrete regulatory standards, funding priorities, and evaluation indicators. The policy–normative mapping presented and detailed in Supporting Information S4: Table 4 provides a framework to guide this translation across diverse health system contexts.

## 7 Conclusion

This study demonstrates that violence against nurses in 18 Latin American and the Caribbean countries during the COVID‐19 pandemic was not an episodic or incidental phenomenon, but a structural expression of longstanding weaknesses in health systems, exacerbated under crisis conditions. Physical aggression, symbolic violence, social stigmatization, and ethical–political suffering emerged as interconnected processes that exposed nurses as the most visible and vulnerable actors within collapsing systems of care.

The findings indicate that nurses' suffering did not result from individual shortcomings but from institutional arrangements that normalized precarious working conditions while shifting responsibility for systemic failures onto nursing professionals directly involved in patient care. In this sense, violence and moral injury functioned as mechanisms of structural blame, eroding professional legitimacy and ethical integrity.

At the same time, narratives of recognition, empathy, and solidarity reveal the persistence of ethical commitment and relational strength within nursing practice, even under extreme adversity. These counter‐narratives highlight nursing not only as a profession affected by crisis, but as a moral and organizational resource for health systems.

Taken together, the results underscore that protecting nurses from violence and moral injury is not optional or secondary, but a prerequisite for resilient, ethical, and sustainable health systems. The lessons drawn from the Latin American and Caribbean countries' experience during COVID‐19 reveal persistent structural conditions that continue to shape violence against nurses beyond emergency contexts, demanding a shift from reactive responses to sustained structural and preventive approaches in nursing governance.

## Use of Artificial Intelligence

During the preparation and revision of this manuscript, the authors used ChatGPT (OpenAI) solely to assist with language editing, improving clarity, grammar, and academic writing. All scientific content, methodological decisions, data analysis, interpretation of findings, and final approval of the manuscript were performed by the authors, who take full responsibility for the accuracy and integrity of the work.

## Author Contributions

Conceptualization: A.S. Methodology: A.S. Investigation: J.L.G.d.S., J.J.‐S., J.I.R.‐L., S.M.A.‐F., M.G.C.‐H., G.C.‐V., P.A.D., and J.A.G.G.‐C. Data curation: L.A.C.T., M.A.C.S., M.G.d.R.M., J.I.R.‐L., M.G.B., and M.G.P. Formal analysis: L.A.C.T., M.A.C.S., M.G.d.R.M., M.G.B., and M.G.P. Supervision: J.L.G.d.S. and A.S. Writing – original draft: L.A.C.T., M.A.C.S., M.G.d.R.M., and J.I.R.‐L. Writing – review and editing: L.A.C.T., J.L.G.d.S., M.A.C.S., M.G.d.R.M., J.J.‐S., J.I.R.‐L., S.M.A.‐F., M.G.C.‐H., G.C.‐V., P.A.D., M.G.B., M.G.P., J.A.G.G.‐C., and A.S. All authors approved the final version of the manuscript and agree to be accountable for all aspects of the work.

## Ethics Statement

The parent study was approved by the New York University Institutional Review Board (IRB‐FY2020‐4440), the institution that established the Global Consortium of Nursing & Midwifery Studies (GCNMS) and served as the IRB of record for all participating country partners. Costa Rica obtained additional approval from the National Ethics Committee (CEC‐468‐2021). All participants provided electronic informed consent before completing the survey. The study was conducted in accordance with the principles of the Declaration of Helsinki.

## Conflicts of Interest

The authors declare no conflicts of interest.

## Supporting information


Supporting File 1

Supporting File 2

Supporting File 3

Supporting File 4

## Data Availability

The data that support the findings of this study are available from the corresponding author upon reasonable request. The data are not publicly available because they contain information that could compromise participant confidentiality and are subject to ethical and consortium data‐sharing agreements.
