# Supplementary material for: Violence, Stigma, and Moral Injury in Nursing During the COVID‐19 Pandemic: A Qualitative Analysis From 18 Countries in Latin America and the Caribbean
Source: Nurs Inq. 2026 Aug 2;33(4):e70153. doi: 10.1111/nin.70153 (PMC13429043; doi:10.1111/nin.70153)
Supplement: Supplementary file 1 — Supporting File 1 [file NIN-33-e70153-s002.docx]

**Supplemental table S1.** Analytical Process of Combining CHD + Reflective Thematic Analysis

| **Stage** | **What was done** | **Decisions/Parameters** | **Evidence/Products** | **Rigor/Control** |
| --- | --- | --- | --- | --- |
| **1. Data extraction and preprocessing** | Extraction of open responses from Qualtrics (.csv); standardization of spelling; removal of incompatible characters; anonymization; contextual translation (ES–PT) by national teams. | Standardization of accents, spelling correction, removal of duplicates, and unification of format across 19 national files; maintenance of terms with semantic value (e.g., relevant hesitations); a minimum density of four words, adopted solely as a technical-operational criterion for inclusion in lexical processing and not as a parameter of interpretive depth. | Individual .txt files for each country; unified corpus with metadata (country, age, gender, workplace). | Audit trails (recorded changes), double manual review, semantic equivalence between languages, and total anonymization guaranteed by Qualtrics. |
| **2. Construction of the corpus in IRaMuTeQ** | Unification of the 19 files, segmentation into UCEs, and automatic lexical processing. | Segment into blocks of approximately 40–50 words; exclude forms with a frequency of 3 or less; ensure a lexical utilization rate of at least 75%; use a minimal stoplist; do not organize by country. | Report of forms and occurrences; utilization rate; complete lexical matrix. | Reproducibility of the corpus, recording of parameters used, and inter-country standardization. |
| **3. Descending Hierarchical Classification (DHC)** | Statistical analysis to identify co-occurrence patterns and lexical classes. | Automatic seed; chi-square association; stable partition into five classes (C1–C5). | Dendrogram; percentage distribution of UCEs; lists of typical words by class; export of typical and atypical UCEs. | Parameters reported in full; solution stability; internal consistency and external heterogeneity confirmed. |
| **4. Descriptive naming of classes** | Initial labeling is based on typical vocabulary and semantic centrality. | Noun + focus rule; provisional labels for C1–C5 correspond to lexical content (no thematic interpretation yet). | Preliminary class dictionary: mapping of semantic nuclei. | Terminological consistency; traceability between vocabulary and label; no interpretive inference at this stage. |
| **5. Interpretive reading of UCEs** | Iterative reading of typical and atypical UCEs; formation of initial codes; identification of tensions, dissonances, and emotional nuances. | Inductive approach; maintenance of discordant cases; preservation of heterogeneity by country; focus on ethical, emotional, and organizational dimensions. | Code notebook: Class → Ideas → Codes Matrix; dated notes. | Confirmability through recording interpretations, researcher reflexivity, and textual triangulation. |
| **6. Construction of themes (Reflective Thematic Analysis)** | Integration of classes into broader narrative processes; inductive grouping. | Integrative reorganization: (C1 + C3) → Theme 1; (C2 + C4) → Theme 2; C5 → Theme 3. Titles should be in the form of a process: violence, stigma, and ethical-political suffering. | Three main themes and emerging subthemes; Theme–Classes–Evidence matrix. | Peer debriefing among authors; internal and external coherence; interpretive sufficiency for thematic construction, considering the stability of lexical classes and the recurrence of narrative patterns. |
| **7. Highlighting findings** | Selection of typical and atypical quotes, geographic variation, and alphanumeric identification. | Criteria: regional diversity, illustrative power, lexical fidelity; anonymized codes. | Analytical table with typical and atypical UCEs; results text organized by themes. | Credibility (participants' voices), audit trail of choices, intentional variation of sources. |
| **8. Theoretical and analytical integration** | Connection of themes with international literature on violence, stigma, moral distress, and systemic collapse. | Convergence and divergence criteria; articulation among structural, relational, and identity levels. | Discussion section: explanation of structural and symbolic mechanisms. | Theoretical coherence, alignment between data and literature, and non-speculative interpretation. |
| **9. Final interpretative synthesis** | Consolidation of findings into an integrated narrative: direct violence leads to stigma, which results in ethical-political suffering. | Analysis of regional transversality, with emphasis on structural vulnerabilities and transnational patterns. | Conclusions, implications for policy, practice, and training, and recommendations. | Cross-check themes, classes, and citations; ensure interpretive closure is consistent with the corpus. |
