# Supplementary material for: Violence, Stigma, and Moral Injury in Nursing During the COVID‐19 Pandemic: A Qualitative Analysis From 18 Countries in Latin America and the Caribbean
Source: Nurs Inq. 2026 Aug 2;33(4):e70153. doi: 10.1111/nin.70153 (PMC13429043; doi:10.1111/nin.70153)
Supplement: Supplementary file 2 — Supporting File 2 [file NIN-33-e70153-s003.docx]

**Supplementary File 2.**

Table. Sociodemographic and professional characteristics of nurses and midwives who reported experiencing aggression during the COVID-19 pandemic in Latin America and Caribbean.

| **Variable** | **N** | **%** |
| --- | --- | --- |
| **Professional category** |  |  |
| Nursing | 3,686 | 96.0 |
| Obstetrics (midwives) | 165 | 4.0 |
| **Country** |  |  |
| Guatemala | 543 | 14.1 |
| Paraguay | 485 | 12.6 |
| El Salvador | 382 | 9.9 |
| Honduras | 264 | 6.8 |
| Bolivia | 258 | 6.7 |
| Ecuador | 215 | 5.6 |
| Mexico | 206 | 5.3 |
| Chile | 206 | 5.3 |
| Argentina | 202 | 5.2 |
| Costa Rica | 190 | 4.9 |
| Venezuela | 188 | 4.9 |
| Colombia | 182 | 4.7 |
| Dominican Republic | 182 | 4.7 |
| Peru | 159 | 4.1 |
| Brazil | 147 | 3.8 |
| Panama | 24 | 0.6 |
| Puerto Rico | 9 | 0.2 |
| Uruguay | 9 | 0.2 |
| **Age (years)**^#^ | 45.0 ± 16.3 | — |
| **Gender** |  |  |
| Female | 3,122 | 81.1 |
| Male | 531 | 13.8 |
| Other | 195 | 5.1 |
| Prefer not to say | 2 | 0.1 |
| **Length of service (years)** |  |  |
| 0–3 | 712 | 23.4 |
| 4–6 | 535 | 17.5 |
| 7–10 | 478 | 15.6 |
| 11–15 | 487 | 16.0 |
| 16–20 | 320 | 10.4 |
| 21–25 | 223 | 7.3 |
| ≥26 | 302 | 9.9 |
| **Population with which** you work* |  |  |
| Adults (19–64 years old) | 2,193 | 70.5 |
| Children <5 years (mothers/children) | 1,334 | 42.7 |
| Elderly (≥65 years) | 1,224 | 39.2 |
| Children and adolescents (6–18 years) | 883 | 28.3 |
| **Position held at work** |  |  |
| Care professional / direct service | 2,092 | 67.3 |
| Management/Administration | 495 | 15.9 |
| Teaching | 229 | 7.3 |
| Other | 297 | 9.5 |
| **Area of activity** |  |  |
| Intensive care unit | 606 | 19.4 |
| Emergency | 550 | 17.6 |
| Public/community health/health promotion | 522 | 17.0 |
| Medical clinic (includes medical-surgical clinic and oncology) | 415 | 13.3 |
| Obstetrics/maternal and child health | 238 | 7.6 |
| Intermediate care unit / semi-intensive care | 181 | 5.8 |
| Operating room / surgery | 191 | 6.1 |
| Mental health/psychiatry | 37 | 1.2 |
| Long-term care / rehabilitation | 37 | 1.2 |
| Other | 247 | 7.9 |
| Prefer not to say | 89 | 2.9 |
| **Type of service where you work** |  |  |
| Hospital | 2,220 | 71.1 |
| Primary/community/outpatient care | 601 | 19.5 |
| Home care | 35 | 1.1 |
| Professional agency/cooperative | 8 | 0.3 |
| Other | 212 | 6.8 |
| Prefer not to say | 37 | 1.2 |
| **Territorial context of the workplace** |  |  |
| Urban | 2,515 | 80.9 |
| Rural | 365 | 11.7 |
| Peri-urban | 156 | 5.0 |
| Prefer not to say | 77 | 2.5 |
| **Qualification** |  |  |
| Undergraduate (bachelor's degree) | 1,739 | 56.0 |
| Master's | 574 | 18.4 |
| Technical/technologist/diploma/associate degree | 559 | 17.9 |
| Doctorate | 68 | 2.2 |
| Prefer not to say | 173 | 5.5 |
| **Type of employment contract** |  |  |
| Full-time | 1,724 | 55.5 |
| Temporary contract | 587 | 18.8 |
| Part-time (<24 hours/week) | 255 | 8.2 |
| Daily contract / on-call contract | 145 | 4.6 |
| Other | 402 | 12.9 |

^#^ Values are mean ± SD. ***Multiple responses allowed. Percentages may exceed 100%. Totals vary due to missing data.
