# Supplementary material for: Violence, Stigma, and Moral Injury in Nursing During the COVID‐19 Pandemic: A Qualitative Analysis From 18 Countries in Latin America and the Caribbean
Source: Nurs Inq. 2026 Aug 2;33(4):e70153. doi: 10.1111/nin.70153 (PMC13429043; doi:10.1111/nin.70153)
Supplement: Supplementary file 3 — Supporting File 3 [file NIN-33-e70153-s004.docx]

**
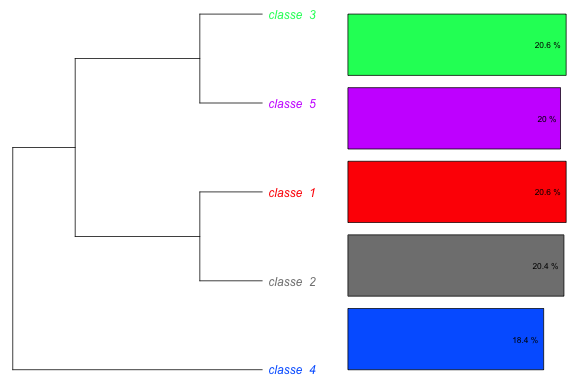
**

**Supplemental Information S3** – Percentage distribution of UCE by lexical class of the CHD. (image generated by Iramuteq software)
