# Supplementary material for: Violence, Stigma, and Moral Injury in Nursing During the COVID‐19 Pandemic: A Qualitative Analysis From 18 Countries in Latin America and the Caribbean
Source: Nurs Inq. 2026 Aug 2;33(4):e70153. doi: 10.1111/nin.70153 (PMC13429043; doi:10.1111/nin.70153)
Supplement: Supplementary file 4 — Supporting File 4 [file NIN-33-e70153-s001.docx]

**Supplemental Table S4.** Detailed alignment of empirical findings and policy implications with the WHO Pandemic Agreement and WHO/ICN frameworks

| **Empirical findings (derived from Table 1)** | **Policy implications** | **WHO Pandemic Agreement (article and rationale)** | **Alignment with WHO / ICN policy frameworks *** |
| --- | --- | --- | --- |
| **Direct, symbolic, and institutional violence against nurses**, manifested through verbal aggression, moral blame, and organizational neglect during system collapse (Themes 1 and 3) | Development and enforcement of institutional policies for prevention, reporting, and accountability regarding workplace violence, particularly during health emergencies | **Article 7** – Requires protection and sustainability of the health workforce, including safe working environments and institutional responsibility | World Health Organization, International Labour Organization, International Council of Nurses, & Public Services International (2002); **International Council of Nurses (2017)** |
| **Ethical-political suffering and moral injury**, resulting from structural scarcity, impossible care demands, and institutional betrayal (Theme 3) | Integration of psychosocial support, ethical consultation, and moral injury prevention strategies into workforce and emergency policies | **Article 6** – Emphasizes strengthening health systems and workforce capacity, including mental health and well-being of health workers | World Health Organization (2022); International Council of Nurses (2020) |
| **Institutional exclusion of nurses from decision-making processes**, leading to moral domination and structural blaming (Themes 1 and 3) | Expansion of nursing leadership, governance roles, and formal participation in crisis management and resource allocation | **Article 7** – Highlights the need for inclusive workforce governance and leadership development | World Health Organization (2021); International Council of Nurses (2020a) |
| **Social stigma, fear of contagion, and symbolic violence**, extending beyond workplaces into families and communities (Theme 2) | Implementation of coordinated public communication and anti-stigma strategies to counter misinformation and protect professional identity | **Article 18** – Addresses risk communication, public trust, and community engagement during health emergencies | World Health Organization (2020); International Council of Nurses (2020b) |
| **Persistent structural vulnerability of the nursing workforce in future Heath crises** (All themes) | Pandemic preparedness frameworks that position nursing workforce protection as a core ethical and operational pillar | **Articles 6, 7, and 19** – Focus on preparedness, workforce protection, and international cooperation | World Health Organization (2021); International Council of Nurses (2022) |

**Note.** Empirical findings are derived from the reflective themes presented in Table 2. Policy alignment reflects a normative interpretation of the WHO Pandemic Agreement and selected World Health Organization and International Council of Nurses policy documents

***REFERENCES**

International Labour Organization, International Council of Nurses, World Health Organization & Public Services International. (2002) Framework Guidelines for Addressing Workplace Violence in the Health Sector. ILO, Geneva.

International Council of Nurses. (2017). Position statement: Prevention and management of workplace violence. <https://www.icn.ch/sites/default/files/inline-files/PS_C_Prevention_mgmt_workplace_violence.pdf>

International Council of Nurses. (2020a). ICN position statement: Nurses’ well-being and mental health. <https://www.icn.ch/sites/default/files/2023-04/PS_A_Mental%20Health_1.pdf>

International Council of Nurses. (2020b). COVID-19 and the international supply of nurses. <https://www.icn.ch/resources/publications-and-reports/covid-19-and-international-supply-nurses>

International Council of Nurses. (2022) Violence against health care: Current practices to prevent, reduce or mitigate violence against health care. ICN, Geneva. Available at: <https://www.icn.ch/resources/publications-and-reports/violence-against-health-care-current-practices-prevent-reduce-or>

World Health Organization. (2020). Risk communication and community engagement (RCCE) action plan guidance: COVID-19 preparedness and response. <https://iris.who.int/bitstream/handle/10665/331513/WHO-2019-nCoV-RCCE-2020.2-eng.pdf>

World Health Organization. (2021). *Global strategic directions for nursing and midwifery 2021–2025*. <https://www.who.int/publications/i/item/9789240033863>

World Health Organization. (2022). World mental health report: Transforming mental health for all. <https://www.who.int/publications/i/item/9789240049338>
